# Meningioma in mature cystic teratoma of the ovary: clinical and computed tomography findings

**DOI:** 10.1186/s40644-020-0291-8

**Published:** 2020-02-05

**Authors:** Yi-yang Liu, Pan Liang, Jiang Ji, Kui-sheng Chen, Li-ming Li, Jian-bo Gao, Liu-liang Yong

**Affiliations:** 1 Department of Radiology, The First Affiliated Hospital of Zhengzhou University, Zhengzhou, 450052 Henan Province China; 2 Department of Radiology, General Hospital, Ningxia Medical University, Yinchuan, 750004 China

**Keywords:** Mature cystic teratoma, Meningioma, Ovary, Computed tomography

## Abstract

**Background:**

Mature cystic teratoma (MCT) with meningioma of the ovary is a very rare benign tumor. There is only 3 reports of this disease until June 2019. The aim of the present study was to describe a ovarian mature cystic teratoma containing meningioma and nests of neuroblasts in a 15-year-old girl.

**Methods:**

The method used in the present study consists of description of the clinical history, image lab features, and pathological result.

**Results:**

The patient complained of a 2-month history of irregular vaginal bleeding. Abdominal computed tomography (CT) showed a large oval cystic-solid mass with septations and fat density shadow, in abdomen pelvic cavity. The cystic part was the main component in the mass. The tumoral solid parts and its internal division could be seen intensified from slight to moderate on contrast-enhanced CT images compared with those on precontrast images, and the solid parts showed heterogeneous enhancement. Neighbouring intestinal tract and the uterus displaced by compression. The pathological examination confirmed the diagnosis.

**Conclusions:**

The clinical feature of ovarian mature cystic teratoma with meningioma includes a lack of specificity. Only meticulous recording of the gross features, histopathological examination including immunohistochemistry and supportive clinical and radiological findings to arrive at a correct diagnosis in case of unconventional tumours. If necessary, preoperative puncture can be performed.

## Background

Mature cystic teratomas are the germ cell tumor deriving from primordial germ cells and composes of tissues arising from endoderm, mesoderm, and ectoderm in terms of histology [1]. Malignant elements or the secondary benign tumors in MCTs can also be seen [1, 2]. However, the incidence of their co-exist in MCTs is extremely rare. An extensive literature search has revealed only three cases of mature cystic teratoma with meningioma of the ovary reported in medical literature, two of which are in English. And the majority of them have focused on the pathology and clinical manifestation; previous reports have not described the radiological appearance of the similar tumor [3, 4]. Moreover, to the best of our knowledge, meningioma co-existing with the nests of neuroblasts in the same mature cystic teratoma has not been reported. Due to the low incidence, understanding of the pathogenesis of other tumors or/and lesion arising within MCTs remains rudimentary, with each new case potentially bringing additional insight [5]. Furthermore, it is significant to distinguish between benign and malignant components that arise in teratomas because of the therapeutic and prognostic importance of their identification. In the present study, we discussed the computed tomography (CT) findings, pathologic characteristics and the clinical features of a rare case of MCTs with reviewing the relative references.

## Methods

A case report of meningioma and nests of neuroblasts arising from mature cystic teratoma of the ovary in an adolescent female was presented. We searched PubMed, Medline, Google Scholar, Chinese Biomedicine Database, and the China Journal Full Text Database without language restriction. The search terms included (meningioma [MeSH]) AND Ovarian teratoma [MeSH]). The demographic, clinical features and imaging findings of both the newly described and previously reported cases are summarized and discussed.

## Results

### Our case description

#### Clinical characteristics

A 15-year-old girl presented with a 2-month history of irregular vaginal bleeding. She complained of abdominal distension and progressive aggravation, occasional abdominal pain. On examination, a large mass was found in abdomen pelvic cavity.

Physical examination revealed the abdominal cavity had giant mass with the upper boundary between the belly button and the xiphoid process. The margin of the mass was clear and the activity was poor. Laboratory blood tests were as follows: platelet count 366 × 10^9^/L, hematocrit count 0.348 L/L, and fibrin degradation products count 18.34 mg/L. No abnormalities were revealed except for cancer antigen 125 (121.20 U/mL) and postmenopausal roman index in (39.50%) the elevated tumor markers.

#### Imaging findings

**Fig. 1:**
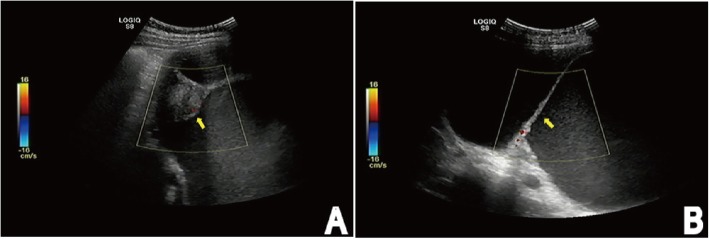
US shows a huge pelvic cavity solid-cystic mass with separation (arrow) (**b**), and an echoic nodule (arrow) in the wall (**a**). The mass is mainly composed of cystic components with poor sound transmission, and dense light spot echo could be detected. (CDFI) shows Punctate blood flow signal in the tumoral nodule, wall and internal division

Ultrasonography (US) displayed a huge pelvic cavity solid-cystic mass measuring 250 × 195 × 86 mm with separation, and an echoic nodule in the wall. The solid component measuring 52 × 32 mm. The mass was mainly composed of cystic components, which were poor in sound transmission, and dense light spot echo could be detected. Color Doppler flow imaging (CDFI) showed Punctate blood flow signal in the tumoral nodule, wall and internal division (Fig. 1a, b). Finally, ultrasound diagnosis was cystic and solid mass in the abdomen pelvic cavity.

**Fig. 2:**
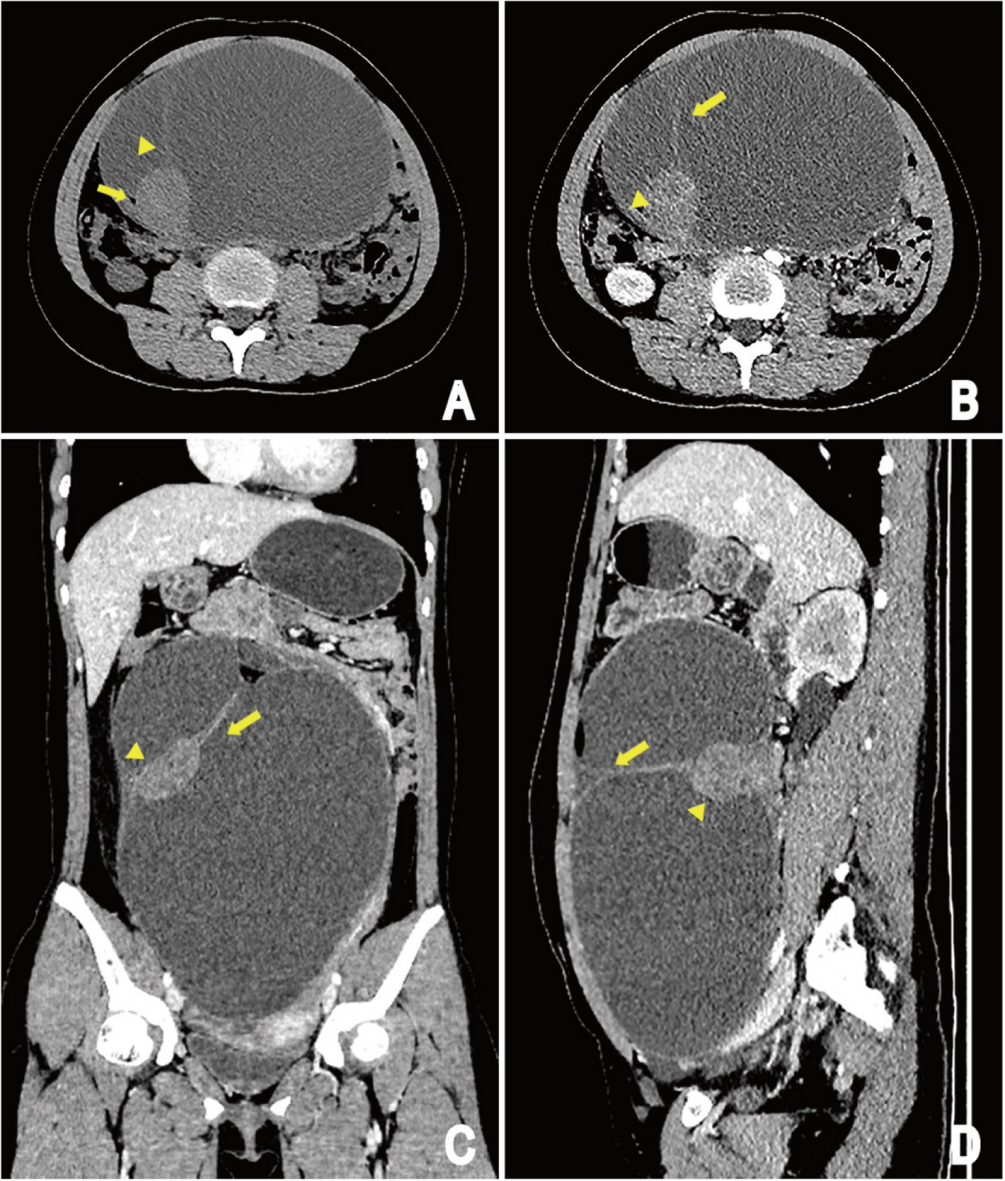
CT images of patient: Unenhanced CT image shows a large oval cystic-solid mass in abdomen pelvic cavity, originating from the left ovary, the margin of the mass is clear and the cystic part is the main component in the mass; The round-like solid component shows inhomogeneous density shadow, CT value is about 39 HU, with smooth edge yet. The cystic part has internal division change (arrowhead) and fatty lesions (arrow) (**a**). The tumoral solid components (arrowhead) and its internal division (arrow) could be seen intensified from slight to moderate on contrast-enhanced CT images compared with those on precontrast images, and the solid component shows heterogeneous enhancement. Adjacent organs such as the intestine, the uterus displaced by compression (**b**, **c** and **d**). (**b**) Arterial phase of contrast enhancement image; (**c** and **d**) portal phase of contrast enhancement image

CT scan of the abdomen revealed a large oval cystic-solid mass (19.6 × 12.5 × 26.3 cm) with septations and fat density shadow in abdomen pelvic cavity. The cystic parts was the main component in the mass and the margin of the solid parts (3.8 × 4.1 × 3.6 cm) was clear (Fig. 2a). The tumoral solid parts and its internal division could be seen intensified from slight to moderate on contrast-enhanced CT images, with the solid parts showing heterogeneous enhancement. Neighbouring intestinal tract and the uterus displaced by compression (Fig. 2b, c and d).

#### Pathological features and follow-up

Laparoscopic left salpingo-oophorectomy and Separation of pelvic adhesions were performed. Surgical exploration revealed a large cystic mass located at the left ovary. Fat, hair, scalp-like tissue could be seen when the tumors were dissected, with dark red and bloody fluid inside, as well as a solid nodule.

On gross description, the Pelvic cavity showed a multiloculated cystic mass measuring 20 × 13 × 27 cm in largest dimension, with gray brown solid areas measuring 4 × 4.2 × 3.9 cm. The uterus, bilateral tubes, and right ovary were unremarkable. There was hair-like sample with a diameter of about 1.1 cm. The thickness of the inner wall was 0.4–0.5 cm, and the inner wall was smooth.

**Fig. 3:**
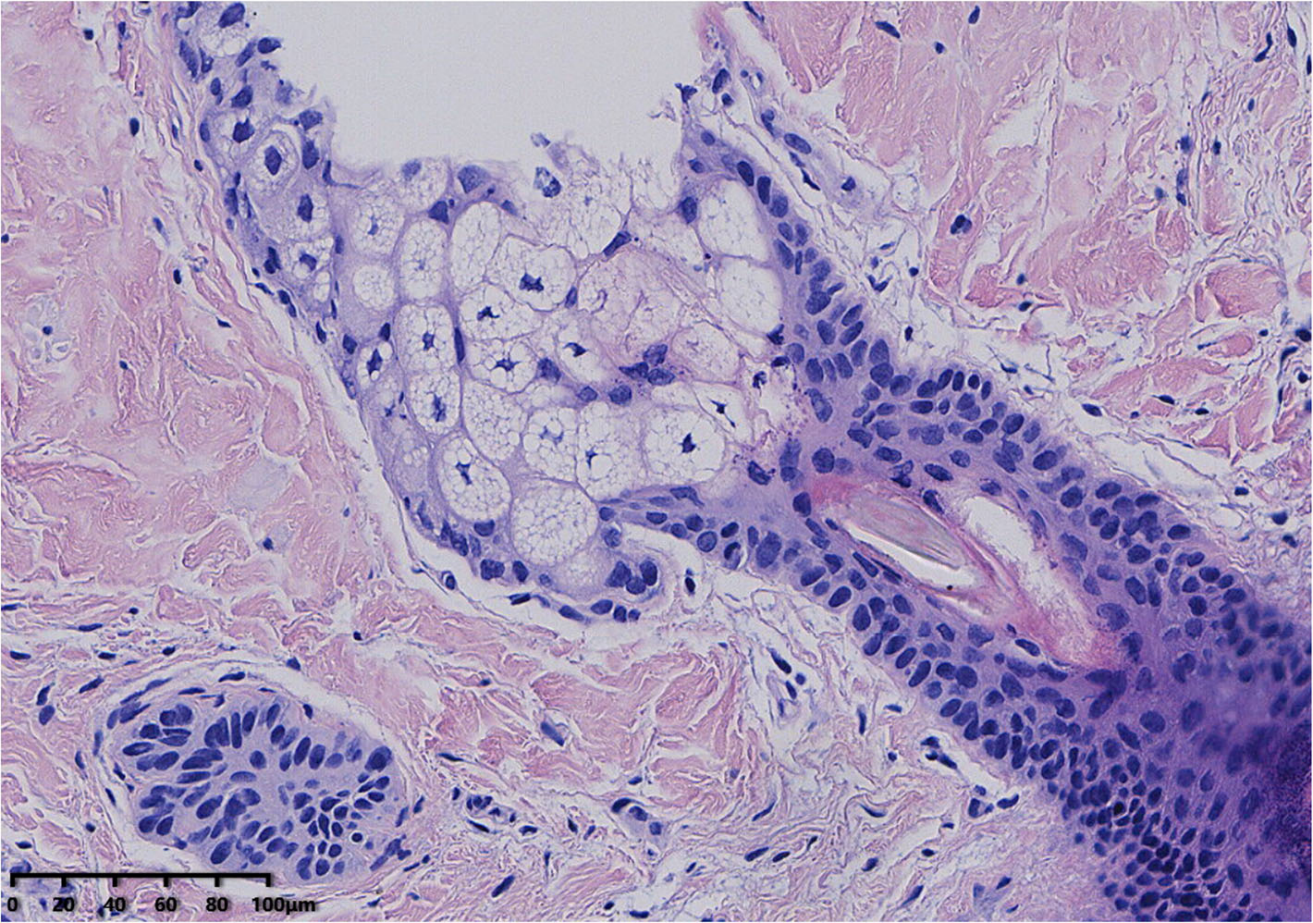
Microscopic examination of the cystic areas. Image shows sebaceous glands, hair follicle, and squamous epithelium. (HE, × 200)

**Fig. 4:**
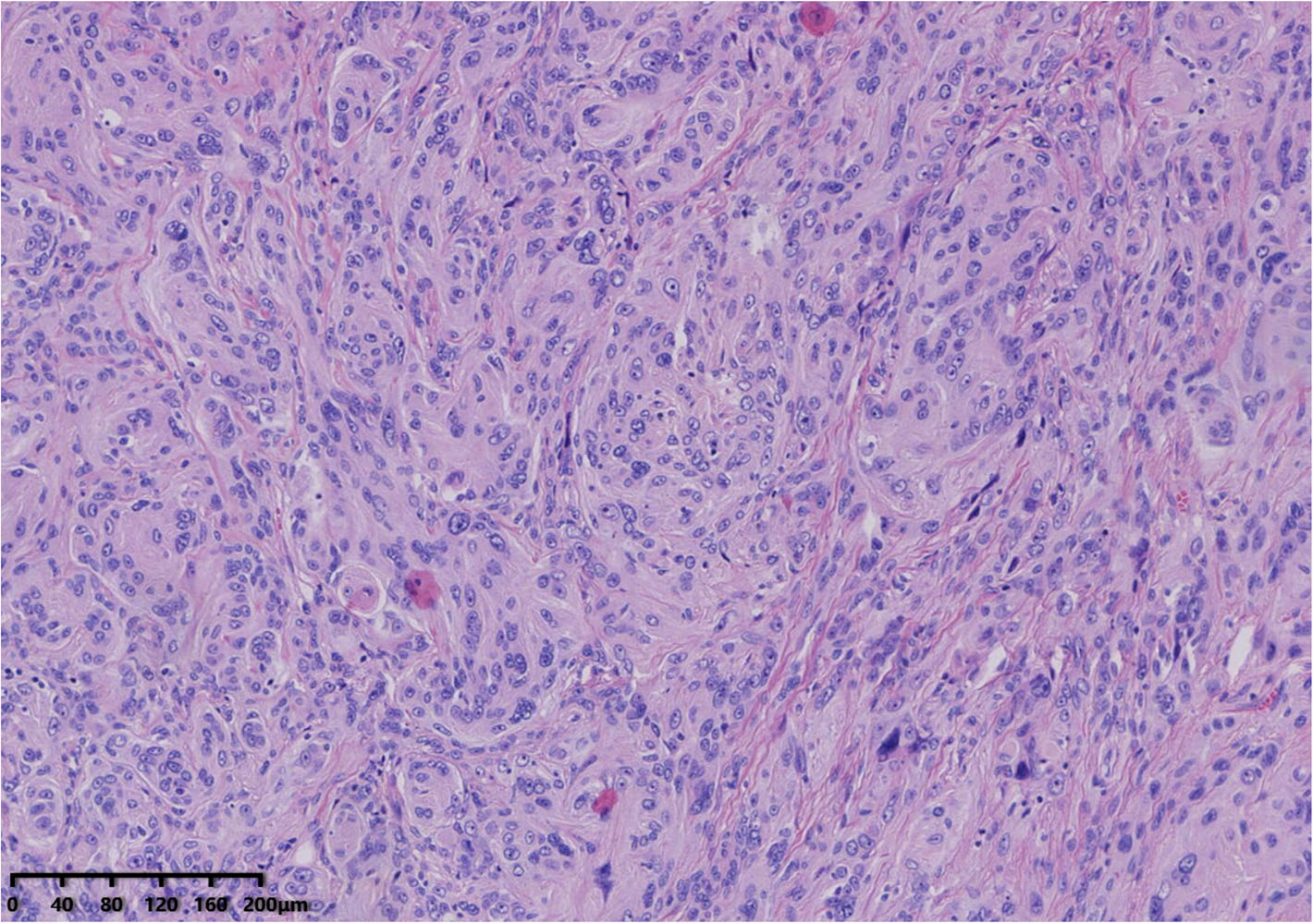
Histological appearance (hematoxylin and eosin stain) of Sections from the solid areas. Histological appearance of the solid areas reveals spindle cells in lobular growth pattern, syncytium-like appearance due to poorly defined cell borders, scattered clear nuclear holes, and occasional intranuclear pseudoinclusions, suggesting a meningothelial meningioma. (H&E, × 100)

Microscopic examination of the cystic areas shown sebaceous glands, hair follicle, and squamous epithelium (Fig. 3). Sections from the solid areas revealed spindle cells in lobular growth pattern, syncytium-like appearance due to poorly defined cell borders, scattered clear nuclear holes, and occasional intranuclear pseudoinclusions, suggesting a meningothelial meningioma (WHO grade I) (Fig. 4). These cells were immunoreactive for somatostatin receptor (SSTR2), Progesterone receptor (PR), epithelial cadherin (E-cadherin) but negative for glial fibrillary acidic protein (GFAP). The labeling index of Antigen KI67 (Ki-67) was less than 10%. The meningioma component was found entirely within the confines of the tumor mass.

**Fig. 5:**
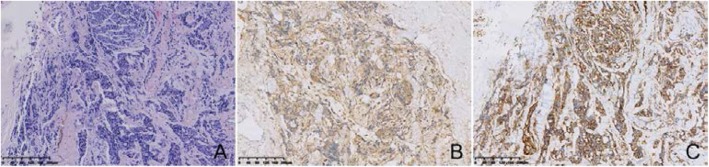
Microscopic and immunohistochemical features of the local cyst wall. The tumour cells was small with a round or polygonal, darkly staining nucleus with signs of division (H&E, × 100) (**a**). Immunohistochemical staining supported Source of neuroblasts with positive staining for S-100 protein (**b**) and CD56 (**c**)

In addition, a few epithelioid cell nests were found on the wall of the cyst, and the cell heterosexuality was obviously accompanied by necrosis. The tumour cells was small with a round or polygonal, darkly staining nucleus with signs of division (Fig. 5a). Immunohistochemical staining supported Source of neuroblasts with positive staining for S-100 protein and CD56 (Fig. 5b and c).

Final pathological diagnosis of this tumor was a mature ovarian cystic teratoma containing meningioma and nests of neuroblasts.

After 4 months of follow-up by ultrasonography, no complication or recurrence was observed.

## Literature review

We initially identified 3 relevant items in PubMed, Medline, Google Scholar, Chinese Biomedicine Database, and the China Academic Journals Full-Text Database. Publication dates ranged from 1968 to June 2019. After reviewing each publication, we selected 2 original studies in English. The characteristics of these patients with ovarian teratoma containing meningioma was shown in Table 1.

**Table 1:** Clinical and imaging findings analysis of mature cystic teratoma with meningioma in 3 patients

Case	Age,y	Size, cm	solid areas			Trentment	Prognosis	Other component
			Border(I/R)	Size, cm	Enhance			
1 Takeshima et al. [3]	60	10 × 10 × 8	NE	3x3x2	NE	RSO	NR	NO
2 Shramana et al. [4]	45	12.4x11x9.5	NE	4 × 3.2 × 3	NE	HBSO	NR	NO
3 present case	15	19.6 × 12.5 × 26.3	Regular	3.8 × 4.1 × 3.6	(+)	LSO	NR	Nests of neuroblasts

*RSO* Right salpingo-oophorectomy, *HBSO* hysterectomy with bilateral saplingo-oophorectomy, *LSO* Left salpingo-oophorectomy, *NE* no evaluation, *NR* no recurrence

## Discussion

MCTs are common ovarian neoplasms characterized by the presence of elements of the 3 germ layers. They are diverse neoplasms with a wide range of epidemiology, histological characteristics, and biological behavior. A variety of tissue types derived from one or more germ cell layers can be observed microscopically, but the most common elements consist of cartilage, tissue of the central nervous system, and epithelium including respiratory, gastrointestinal, and cutaneous systems [6–8]. Tumor or benign and malignant components can also be observed. To date, the secondary benign tumors including mucinous cystadenomas, compound nevus, blue nevus, prolactinoma, ACTH-producing pituitary adenoma, epithelioid hemangioma, sebaceous adenoma and benign skin adnexal tumor arising in mature cystic teratomas of the ovary have been described [3].

Teratomas containing meningiomas have been found only in the ovary and testes [2–4, 9]. In addition, teratoma containing neuroblastoma or immature neuroepithelial elements has also been reported in ovary. Among teratoma, secondary benign and malignant neoplasms or elements may arise from the tissues constituting teratomas. The histogenesis of such case of meningioma can be considered as a benign or malignant transformation of the components of mature cystic teratoma. The arachnoid cells of some organs can be considered as the source of meningiomas, such as the dura, nasal and paranasal sinuses, skin, lungs, mediastinum, and peripheral nerves [3]. Therefore, arachnoid cells of some tissues in teratomas have been the origin of this tumor. In addition, our case reconfirmed that immature neuroepithelial elements have been present in the some mature teratoma [10].

Teratoma with meningioma of the reproductive organs was originally described by Takeshima et al. [3] in 2004. Teratoma containing immature neuroepithelial elements in the reproductive organs was firstly described by McCullough et al. in 1963. Nevertheless, malignant transformation of teratoma components only can be seen in 3–6% of metastatic germ cell tumors. Secondary benign neoplasm in teratomas is also extremely rare [2]. In a review of published work until May 2019, we found only two English case reports about meningioma in a mature cystic ovarian teratoma [3, 4].

The mean age of the patients with MCT was 37.5 years. The patient’s age in the present study was only 15 years compared to 52.5 years in the mean age of the two previously published patients [11]. The clinical manifestation was not typical, where by it can be hidden for a long time [3]. Pain and enlargement of abdomen was the most common symptoms. Irregular vaginal bleeding may be caused by neuroendocrine dysfunction regulating reproduction. Serum tumor markers played an important role in the differential diagnosis of malignant transformation arising from MCT and MCT. Kikkawa et al. [11] reported that there were significant differences levels of squamous cell carcinoma antigen (SCC), CA125, CEA, and CA19–9 between MCT and squamous cell carcinoma arising from MCT. Although this case was a mature cystic teratoma, CA125 was significantly elevated, about 121.20 U/ml. From a clinical point of view, CA125 was also significantly elevated in benign ovarian tumors, but usually less than 200 U/ml.

Due to the rarity of this tumor, imaging has not made an accurate diagnosis or even detailed description. Ultrasonography is the most common, economical, and simplest method for clinical diagnosis of ovarian cystic teratomas. Specifically, the US findings of mature cystic teratomas are various. For instance, a cystic lesion with a densely echogenic tubercle projecting into the cyst lumen, or with the sebaceous material and hair in the cyst cavity, it presents as a diffusely or partially echogenic mass with the echogenic area normally showing sound attenuation, or a cyst cavity with multiple thin echogenic bands. However, definite diagnosis may be limited by only assessing the internal structure and echogenicity of ovarian mass. Therefore, this diagnostic option is not appropriate in the present study [12, 13]. In addition, CT seems to be the best modality to assist in the diagnosis of ovarian cystic teratoma [14], it has been commonly used to study the possible communication between the mass and the ovary, adjacent organs, and to further description of the mass.

In the present case, the tumor manifested as a multiloculated cystic mass with mild to moderate degree enhancement of solid part and internal division in the cyst, and fat components can be seen, without calcification. The histopathological examination shows that the tumor consists of soft tissue composed of spindle cells and a cystic space, with the thin-walled cystic lesion has connective septa covered by fibrocyte. Moreover, a few epithelioid cell nests are founded in the wall of the cyst. Generally, there is a raised protuberance in the cyst cavity of MCT, just like the soft tissue component in this case, which is called the Rokitansky protuberance [12]. It is important to recognize malignant transformation of Rokitansky nodule that arise in teratomas because of related to the prognosis of patients, especially when Rokitansky nodule are tumors. Patients in whom the malignant component is localized to the organ of origin do well, but patients in whom the non-germ cell component metastasizes do poorly [15].

For all these reasons, we are committed to differentiating secondary benign component from malignant component arising in ovarian cystic teratoma on CT findings.

The imaging manifestations of secondary malignant tumors in cystic teratomas have been discussed previously. Imaging findings of the tumor usually presented as: larger than 9.9 cm of the largest diameter, the presence of enhancing soft tissue components, an obtuse angle between the soft tissue components and the inner wall of the cyst, as well as extracapsular tumor growth with extension into adjacent structures or disseminated metastasis [13]. Buy et al. [14] reported two cases of malignant transformation of teratomas about CT findings, the suspected cause of the malignancy was a solid mass larger than 5 cm in diameter with irregular borders forming an obtuse angle with the inner wall of the cyst in one case and with uptake of contrast medium in another case. In our case, although the maximum diameter of the soft tissue composition was more than 5 cm as well as the border of the enhancing soft tissue components made an obtuse angle laterally with the cyst wall, the soft tissue appearance is smooth and regular. Finally, according to the WHO classification of tumors of the nervous systems [16], the meningioma constituents is considered to be a benign meningioma (WHO grade I). Therefore, Rokitansky protuberance is an important CT finding.

Ovarian teratoma containing meningioma is a rarely seen tumor. Pure ovarian cysts can be identified by ultrasound alone. Pure ovarian cysts usually have a circular or elliptical clear liquid sound transmission area with clear boundaries and thin, smooth cyst wall with a diameter of 3–8 cm. An acoustic enhancement effect was observed on the back wall and posterior of the cyst. CDFI showed no blood flow in the cyst wall [17]. Nevertheless, the definitive diagnosis of ovarian mature cystic teratoma containing meningioma and nests of neuroblasts depended on a combination of clinical, radiological, and histopathological evidences. Despite mature cystic teratoma containing a low grade meningeoma being a benign disease, there is a possibility of recurrence and malignant transformation. In the terms of secondary malignant transformation arising from mature cystic teratoma. The surgical resection appears to be the most effective treatment, especially when the tumor increases in size and applies pressure on the surrounding tissues. Specific methods including bilateral salpingo-oophorectomy, total hysterectomy, omentectomy and pelvic-paraaortic lymph node dissection. After the operation is completed, radiotherapy or chemotherapy can be performed according to the actual condition of the patient [18] .However, when there is no malignant transformation in MCT, laparoscopic surgery is usually performed [11]. Therefore, knowledge of any possible malignant transformation of a mature teratoma and its relationship to adjacent organs could be valuable for surgical planning [19]. Inaddition, for young women of childbearing age or fertility requirements with pure ovarian MCT, surgical procedures are often used to ovarian cysts decollement and preserve normal ovarian tissue on the affected side. Takeshima et al. [3] reported a 60-year-old woman with microcystic meningioma arising in a mature cystic teratoma of the right ovary, who performed salpingo-oophorectomy. Mandal et al. [4] reported a 45-year-old female with psammomatous meningioma arising in a mature cystic teratoma of the left ovary, who carried out total abdominal hysterectomy with bilateral saplingo-ophorectomy. While recurrence of meningioma in mature cystic teratoma of the ovary had not been reported previously, the benign elements of cystic teratoma can be transformed into malignant elements and malignant epithelium that develop within a pre-existing teratoma can continue to proliferate and clone [4]. Therefore, strict follow-up after surgery is necessary.

## Conclusion

Ovarian mature cystic teratoma containing both meningioma and neuroblasts components is extremely rare, but preoperative diagnosis is very important because it involves the choice of treatment options and prognosis of patients. It is recommended to determine the pathology through preoperative puncture when fat and enhanced soft tissue components are found in cystic tumors during CT examination. Despite it being a benign disease, there is a possibility of recurrence and malignant transformation. Radical surgical resection is needed to treat this tumor and discreetly follow-up with CT or ultrasound is essential.

## Data Availability

Not applicable.

## Abbreviations

ACTH: Adreno-cortico-tropic-hormone
CA125: Carbohydrate antigen 125
CA19–9: Carbohydrate antigen 19–9
CD56: Carbohydrate antigen 56
CDFI: Color Doppler flow imaging
CEA: Carcinoembryonic antigen
CT: Computed tomography
E-cadherin: Epithelial cadherin
GFAP: Glial fibrillary acidic protein
GFAP: Glial fibrillary acidic protein
H&E: Hematoxylin and eosin
Ki67: Antigen KI67
MCT: Mature cystic teratoma
PR: Progesterone receptor
S100: Soluble protein-100
SCC: Squamous cell carcinoma antigen
SSTR2: Somatostatin receptor
US: Ultrasound
WHO: World Health Organization
